# Supplementary material for: Natural History of Sudan ebolavirus to Support Medical Countermeasure Development
Source: Vaccines (Basel). 2022 Jun 16;10(6):963. doi: 10.3390/vaccines10060963 (PMC9228702; doi:10.3390/vaccines10060963)
Supplement: Supplementary file 1 [file vaccines-10-00963-s001.zip › vaccines-1736160-supplementary.pdf]

vaccines

MDPI

Supplementary Materials

Table S1. Summary of individual animal clinical scoring.

Animal ID	Day P.I	D0 (am)	D1 (am)	D2 (am)	D3 (am)	D4 (am)	D4 (pm)	D4 (night)	D5 (am)	D5 (pm)	D5 (night)	D6 (am)	D6 (pm)	D6 (night)	D7 (am)	D7 (pm)	D7 (night)	D8 (am)	D8 (pm)	D8 (night)	D9 (am)	D9 (pm)	D9 (night)	D10 (am)
**655**	Time P.I (hr:min)	0	22:21	47:40	71:10	95:20	102:55	107:11	118:20	126:35	130:40	142:05	148:40	155:03	165:40									
	Score	0	0	0	0	7	5	4	9	11	11	10	10	10	15									
**656**	Time P.I (hr:min)	0	22:21	47:40	71:10	95:20	102:55	107:11	118:20	126:35	130:40	142:05	148:40	155:03	165:40	174:10								
	Score	0	0	0	0	0	2	1	3	6	6	4	8	8	14	19								
**778**	Time P.I (hr:min)	0	22:21	47:40	71:10	95:20	102:55	107:11	118:20	126:35	130:40	142:05	148:40	155:03	165:40									
	Score	0	0	0	1	3	3	3	7	7	7	7	9	9	18									
**837**	Time P.I (hr:min)	0	22:21	47:05	70:35	95:00	102:20	106:36	117:45	126:05	130:05	141:30	148:12	154:28	165:05	173:37	179:48	189:45	196:35	202:42	213:30			
	Score	0	0	0	2	3	5	5	8	8	8	10	10	10	8	10	10	13	13	12	17			
**882**	Time P.I (hr:min)	0	22:21	47:05	70:35	95:00	102:20	106:36	117:45															
	Score	0	0	0	4	5	7	7	17															
**986**	Time P.I (hr:min)	0	22:21	47:05	70:35	95:00	102:20	106:36	117:45	126:05	130:05	141:30	148:12	154:28	165:05	173:37	179:48	189:45	196:35	202:42	213:30			
	Score	1	0	0	2	4	4	5	6	8	8	9	10	10	10	11	12	14	14	14	15			
**356**	Time P.I (hr:min)	0	22:21	46:10	69:40	94:20	101:25	105:41	116:50	125:15	129:10	140:40	147:20	153:33	164:10	172:45	178:53	188:50	195:40	201:47	212:35	219:10	225:44	236:40
	Score	2	0	0	2	3	4	4	3	5	5	5	5	5	7	9	9	14	10	10	14	14	14	17
**396**	Time P.I (hr:min)	0	22:21	46:10	69:40	94:20	101:25	105:41	116:50	125:15	129:10	140:40	147:20	153:33	164:10	172:45	178:53	188:50	195:40	201:47	212:35	219:10	225:44	236:40
	Score	0	0	0	1	4	4	3	4	6	6	6	6	6	9	12	12	12	12	12	12	14	14	20
**739**	Time P.I (hr:min)	0	22:21	46:10	69:40	94:20	101:25	105:41	116:50	125:15	129:10	140:40	147:20	153:33	164:10	172:45	178:53	188:50						
	Score	0	0	0	1	3	2	4	7	9	9	9	11	10	12	14	15	19						
**895**	Time P.I (hr:min)	0	22:21	45:30	69:00	94:00	100:45	105:01	116:10	124:40	128:30	140:00	146:46	152:53	163:30	172:30								
	Score	1	0	0	2	4	5	5	10	12	12	11	10	10	13	15								
**966**	Time P.I (hr:min)	0	22:21	45:30	69:00	94:00	100:45	105:01	116:10	124:40	128:30	140:00	146:46	152:53	163:30									
	Score	0	0	0	2	8	8	8	10	8	8	10	10	10	17									
**985**	Time P.I (hr:min)	0	22:21	45:30	69:00	94:00	100:45	105:01	116:10	124:40	128:30	140:00	146:46	152:53	163:30	172:30	178:13	188:10	195:00	201:07	211:55	218:30	225:04	236:00
	Score	0	0	0	2	4	4	4	9	9	9	9	9	9	12	12	12	13	13	13	14	13	13	18

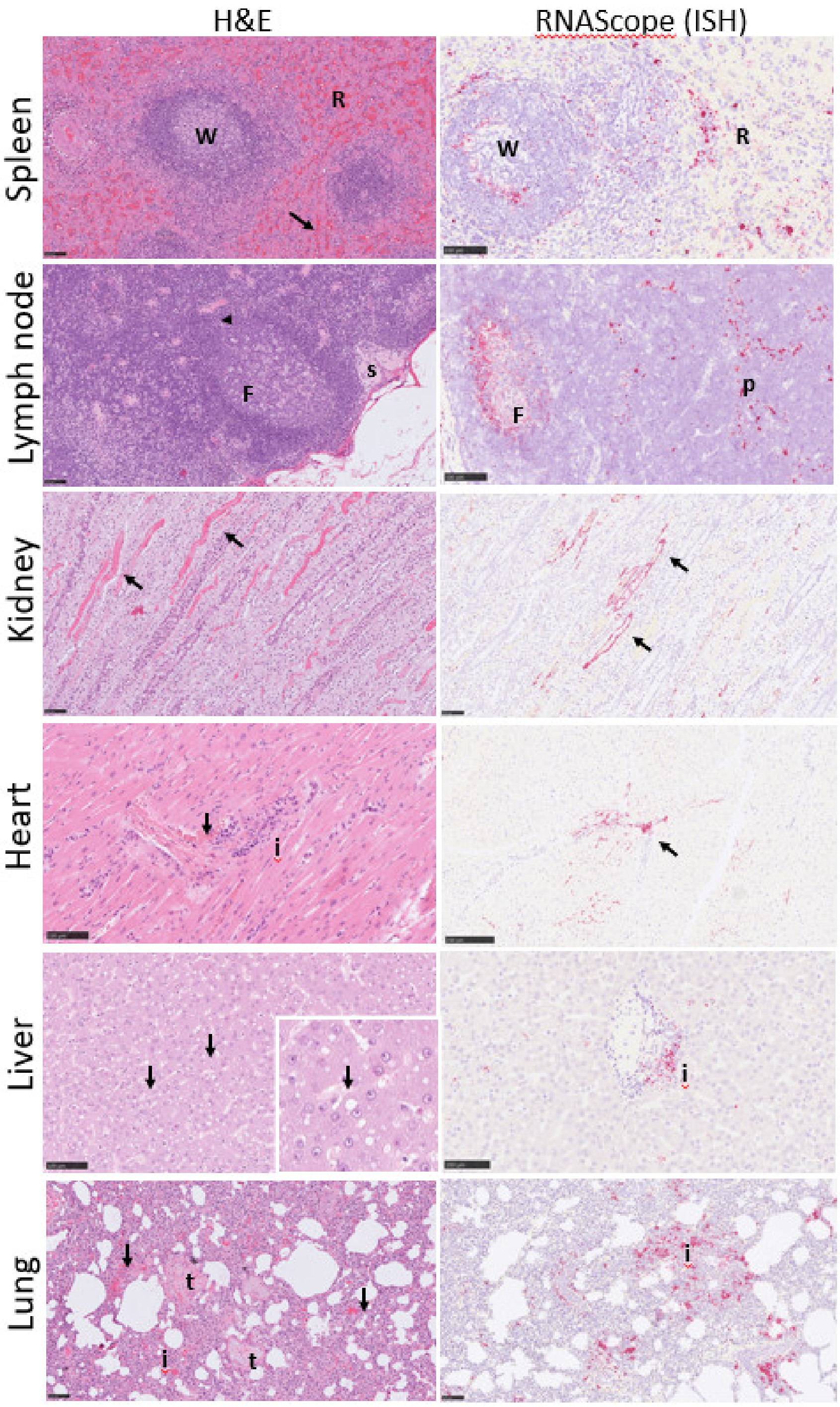

Figure S1. Histopathology. Microscopic lesions (H&E stained) and presence of virus RNA (RNAScope ISH) in spleen, inguinal lymph node, kidney, heart, liver and lung from animal infected with SUDV. Spleen shows lymphoid depletion in white pulp (W) and congestion/hemorrhages (arrow) in the red pulp (R), with presence of abundant virus RNA in both white and red pulp. Lymph node also shows lymph depletion within the follicles (F) and parafollicular tissue, with moderate congestion (arrow) and sinus histiocytosis (S). Virus RNA is observed in all structures. Necrosis is observed in kidney tubules together with presence of fibrin/cell debris casts within the tubular luminae and collecting ducts in the medulla (arrows), showing abundant virus RNA (arrows). Mild inflammatory infiltrates (i) and congestion is observed in the heart (arrow) with small quantities of virus RNA associated to blood vessels (arrow). Small foci of necrosis and hepatocyte cellular degeneration is observed in the liver (arrow, insert) with presence of virus RNA mostly in inflammatory infiltrates within portal areas (i). Foci of inflammatory cell infiltration (i), congestion/hemorrhaging (arrow) and thrombi (t) are present in the lung with virus RNA visible within the inflammatory infiltrates (i) and blood vessel endothelium.

Table S2. Fold change (FC) in the values of the biochemical parameters at the time of euthanasia compared to the baseline in SUDV-challenged animals.

					Fold change from Challenge at the Time of Euthanasia							
		**Animal ID**	**Day PC until death**	**Time PC until Death**	**Urea**	**Creatinine**	**ALP**	**ALT**	**AST**	**CK-NAK**	**Total Bilirubin**	**CRP**
Male	Cabinet 1	655	7	167:05:00	1.6	1.5	2.3	2.7	14.6	5.1	2.3	91.5
		656	7	175:04:00	2.7	4.5	2.9	6	24.2	37.1	5.1	3.9
		778	7	166:50:00	2	1.6	2.6	2.8	13.8	5.7	0.5	36.7
	Cabinet 2	837	9	215:05:00	10.1	15.3	3.2	4.6	21.8	2.2	38.2	31.9
		882	5	120:52:00	1.7	1.9	4.3	21.9	38.2	4.4	3.3	3.9
		986	9	215:05:00	8.8	7.7	2.8	19.2	9.5	6.7	31.5	37.8
		Mean Males (n = 6)			**4.5**	**5.4**	**3**	**9.5**	**20.4**	**10.2**	**13.5**	**34.3**
Female	Cabinet 3	356	10	237:46:00	1.6	1.3	4.5	1.5	2.4	0.2	4.3	5.4
		396	10	238:10:00	2.3	1.9	4.5	3.4	8.6	2.3	2.6	4.4
		739	8	190:25:00	3.7	2.8	5.9	4.2	19.2	5.8	12.9	9.2
	Cabinet 4	895	7	174:57:00	2.5	1.5	3.7	6.6	9.9	3	2.7	6.5
		966	7	168:30:00	2.4	1.9	4.2	5.7	8.4	1.4	2.4	20.5
		985	10	239:00:00	2.7	1.9	2.3	0.9	7.8	1.6	1.8	15.8
		Mean Females (n = 6)			**2.5**	**1.9**	**4.2**	**3.7**	**9.4**	**2.4**	**4.5**	**10.3**
		**Mean (all NHP n = 12)**			**3.5**	**4.1**	**3.6**	**6.6**	**14.9**	**6.3**	**9**	**22.3**
					(light)	≤ FC mean all NHP						
					(medium)	1 < FC mean all NHP ≤ 2						
					(dark)	> 2 FC mean all NHP						

Table S3. Fold Change Hematological Parameters.

					Fold change from challenge at the time of euthanasia							
		**Animal ID**	**Day PI until death**	**Time PI until death**	**WBC**	**Monocytes**	**Neutrophils**	**RBC**	**Hematocrit**	**Hemoglobulin**	**Platelets**	**Lymphocytes**
Males	Cabinet 1	655	7	167:05:00	0.9	0.4	4.7	0.8	0.8	0.8	0.3	0.2
		656	7	175:04:00	0.8	1.2	3.6	0.9	0.8	0.8	0.5	0.5
		778	7	166:50:00	0.8	0.5	1.4	0.8	0.8	0.8	0.2	0.5

	Cabinet 2	837	9	215:05:00	1.4	1.7	5.7	1	0.9	0.8	0.6	0.5
		882	5	120:52:00	1	1	2	0.9	0.8	0.8	0.4	0.4
		986	9	215:05:00	5.3	4	4.5	0.9	0.9	0.9	0.4	2.8
Mean Males (n = 6)					**1.7**	**1.5**	3.7	**0.9**	**0.8**	**0.8**	**0.4**	**0.8**
Females	Cabinet 3	356	10	237:46:00	1.6	0.9	3.1	0.8	0.8	0.8	0.5	0.8
		396	10	238:10:00	1.3	0.4	2.2	0.7	0.7	0.7	0.1	0.4
		739	8	190:25:00	0.6	0.1	1.4	0.8	0.8	0.8	0.3	0.6
	Cabinet 4	895	7	174:57:00	0.5	0.4	0.3	0.9	1	0.9	0.3	0.2
		966	7	168:30:00	0.8	0.4	1.7	0.9	0.9	0.9	0.2	0.4
		985	10	239:00:00	2.8	3.3	5.6	0.7	0.8	0.7	0.7	0.7
Mean Females (n = 6)					**1.3**	**0.9**	**2.4**	**0.8**	**0.8**	**0.8**	**0.4**	**0.5**
**Mean (all NHP n = 12)**					**1.5**	**1.2**	**3**	**0.8**	**0.8**	**0.8**	**0.4**	**0.7**

(light shading)	≤ FC mean all NHP
(medium shading)	1 < FC mean all NHP≤ 2
(dark shading)	> 2 FC mean all NHP

Table S4. Fold change (FC) in the values of the coagulation parameters at the date of euthanasia compared to the baseline in SUDV-challenged animals.

					Coagulation analyses Fold change from baseline at the time of euthanasia			
		**Animal ID**	**Day PI until death**	**Time PI until death**	**ACT**	**PT**	**INR**	**Platelets**
Male	Cabinet 1	655	7	167:05:00	1.6	1.6	1.1	0.3
		656	7	175:04:00	2.2	1.1	1.2	0.5
		778	7	166:50:00	1.8	1.4	1.8	0.2
	Cabinet 2	837	9	215:05:00	2.3	1.1	1.2	0.6
		882	5	120:52:00	2.2	1.3	1.3	0.4
		986	9	215:05:00	2.6	1.8	1.1	0.4
Mean Males (n = 6)					**2.1**	**1.4**	**1.0**	**0.4**
Female	Cabinet 3	356	10	237:46:00	1.3	1.8	1.1	0.5
		396	10	238:10:00	1.3	1	1	0.1
		739	8	190:25:00	2	N/A	1	0.3

Cabinet 4	895	7	174:57:00	1.3	1.9	1	0.3
	966	7	168:30:00	1.8	1	1	0.2
	985	10	239:00:00	1.7	N/A	1.1	0.7
Mean Females (n = 6)				**1.6**	**1.4**	**0.9**	**0.4**
**Mean (all NHP n = 12)**				**1.8**	**1.4**	**1.1**	**0.4**

Shading	Meaning
(lightest grey)	≤ FC mean all NHP
(medium grey)	1 < FC mean all NHP≤ 2
(dark grey)	> 2 FC mean all NHP
N/A	No data from baseline

Table S5. Histopathology scores for each animal and organ. Depletion: lymphoid depletion due to necrosis or apoptosis; H/C: hemorrhages/congestion/hyperemia; Inf/nec: inflammatory infiltrates/necrosis.

	**Spleen**		**Kidney**		**Heart**		**LN**		**Liver**		**Lung**	
**Animal ID**	**Depletion**	**H/C**	**Inf/nec**	**H/C**	**Inf/nec**	**H/C**	**Depletion**	**H/C**	**Inf/nec**	**H/C**	**Inf/nec**	**H/C**
655	3	3	1	2	1	1	3	2	2	0	3	2
656	4	4	3	3	1	2	3	1	1	1	1	1
778	3	3	1	3	0	1	2	1	1	0	1	1
837	4	3	2	2	1	1	3	1	1	1	1	3
882	3	4	1	3	0	1	3	1	1	1	2	1
986	4	4	2	3	1	2	3	2	1	2	1	1
356	2	3	1	2	1	1	2	1	1	0	1	1
396	2	3	2	2	1	1	3	1	1	1	3	3
739	3	2	1	2	1	1	3	3	1	0	2	2
895	2	3	1	1	1	1	4	2	1	0	3	3
966	2	3	2	2	1	2	3	3	2	2	2	3
985	3	3	2	2	1	1	3	2	1	2	4	1

Table S6. Semiquantitative scores for presence of viral RNA in organs by RNAScope.

	Spleen		Kidney		Heart	Lymph node		Liver	Lung
Animal ID	Red pulp	Follicle	Cortex	Medulla		Cortex	Medulla		
655	2	3	2	1	2	4	2	2	3
656	3	3	2	3	2	3	2	3	2
778	2	3	2	2	1	3	3	1	1
837	4	3	3	3	3	3	4	3	2
882	3	2	3	3	1	1	3	2	2
986	4	3	4	4	3	3	3	2	1
356	1	2	2	3	3	2	2	2	1
396	1	2	3	3	3	4	2	2	4
739	2	3	2	2	1	3	4	2	2
895	2	2	3	2	2	4	4	3	3
966	2	1	2	3	2	4	2	3	3
985	2	2	3	3	3	3	3	2	4
